# Parental bereavement and the loss of purpose in life as a function of interdependent self-construal

**DOI:** 10.3389/fpsyg.2015.01078

**Published:** 2015-07-27

**Authors:** Jinhyung Kim, Joshua A. Hicks

**Affiliations:** Department of Psychology, Texas A&M University, College StationTX, USA

**Keywords:** parental bereavement, purpose in life, interdependent self-construal, well-being, adaptation

## Abstract

Children are often inextricably linked to their parents’ hopes and dreams. As such, the loss of a child often represents one of the most traumatic experiences possible. The current research explores how this specific loss relates to one’s sense of purpose in life. We further explore whether the loss of a child is particularly detrimental to one’s sense of purpose for highly interdependent parents. Analyses of parents from the Midlife in the United States data set revealed, as expected, that the loss of child negatively predicts one’s sense of purpose in life, and that this effect is most pronounced for parents high in interdependent self-construal. Potential mechanisms and implications of the present findings are discussed.

## Introduction

People from diverse backgrounds commonly believe that children provide life with meaning and fulfillment (Toulemon, 1996; Stanley et al., 2003). Corroborating these beliefs, a series of studies recently demonstrated that parents experience more positive emotions, less negative emotions, and greater life satisfaction and meaning in life through child-care activities compared to non-parents (Ashton-James et al., 2013; Nelson et al., 2013; see McLanahan and Adams, 1987; Blanchflower and Oswald, 2004; Evenson and Simon, 2005; Kohler et al., 2005; White and Dolan, 2009, for supporting and contradictory findings).

While children often imbue life with purpose and meaning, the loss of child can shake the foundation of one’s existence and detrimentally influence both physical and psychological health. Research shows, for instance, bereaved parents suffer from a wide array of physical and mental illness, including higher incidences of cancer (Li et al., 2002), increased mortality (Li et al., 2003), more severe grief symptoms (Hazzard et al., 1992), post-traumatic stress disorder (Murphy et al., 1999; Spooren et al., 2001), increased anger and hostility (Rando, 1983), shattered personal identity and self-concepts (Neimeyer et al., 2002), and doubts in their world-views (Janoff-Bulman, 1989; Matthews and Marwit, 2003).

Parental bereavement is posited to reduce one’s sense of meaning and purpose in life (Janoff-Bulman and Frantz, 1997). Indeed, a set of empirical data and qualitative investigations demonstrate that bereaved parents often fail to find meaning in the loss experience for an extended period of time after the loss of their child, and that these parents report higher mental distress and lower physical health compared to those who successfully construct a sense of meaning in the loss experience (Lehman et al., 1987; Braun and Berg, 1994; Murphy et al., 2003; Keesee et al., 2008; Lichtenthal et al., 2013; see also Park, 2010 for a review). While prior research has focused primarily on how *situational* meaning and a sense of purpose serve as coping resources following the loss of a child, the present study directly examines how losing a child influences parents’ *global* purpose in life.

Researchers have defined purpose in life as a central, self-organizing life aim that provides a person with a framework for pursuing life goals (McKnight and Kashdan, 2009). Providing an overarching sense of goals and direction in life, purpose in life has been found to be positively associated with happiness and well-being (Ryff, 1989; Byron and Miller-Perrin, 2009; Bronk et al., 2009; Burrow and Hill, 2011). More recently, Hill and Turiano (2014) demonstrated that purpose in life serves to buffer against mortality risk across adulthood using data from the Midlife in United States (MIDUS) sample (see also Boyle et al., 2009). The present research aims to explore the possibility that experiencing the loss of a child may violate parents’ overarching goals and fundamental beliefs about life, and thus reducing their sense of purpose.

A secondary goal of the current research is to explore an unexamined psychological factor that may moderate the effect of loss of child on purpose in life (e.g., Lehman et al., 1987; McIntosh et al., 1993). In the present study, we suggest that individual differences in interdependent self-construal play a pivotal role in the extent to which bereaved parents find it difficult to extract purpose in life from the loss experience. People who hold interdependent self-construals value harmony in social relationships and place close others in the core part of their self-concept, whereas those who hold independent self-construals value autonomy and maintain uniqueness by distancing others from their self-concept (Markus and Kitayama, 1991; Singelis, 1994). The closeness between self and significant others among those high in interdependent self-construal is particularly salient between caregivers and children. For example, among Eastern Asians (i.e., interdependent people), one’s self is predominantly described in terms of their caregivers (Markus and Kitayama, 1991; Bochner, 1994) and, for these people, neural activity that processes self-relevant information does not distinguish between stimuli related to one’s self and one’s mother, for example (Zhu et al., 2007; Chiao et al., 2009). Likewise, children are a central part of the self-concept of parents who possess interdependent self-construals.

Based on the relevant literature on parental bereavement and cultural psychology, we hypothesize that (A) losing a child will detract from one’s overall sense of purpose in life and (B) this relationship will be stronger for parents high in interdependent self-construal than for those low in interdependent self-construal. To test this hypothesis, we employed a longitudinal data set that includes American adult respondents. Specifically, we used the same data set MIDUS that Hill and Turiano (2014) analyzed to demonstrate that purpose in life predicts decreased mortality rates. Although investigations of self-construal are often conducted in a cross-cultural manner (e.g., East vs. West), there is also great variability in self-construal within cultures (Oyserman et al., 2002). The current research focuses on how individual differences in self-construal, rather than cultural differences, moderate the relationship between the loss of a child and purpose in life. From both cross-sectional and longitudinal analyses of the data, we expect that decreased purpose in life by the loss of a child would be more pronounced for parents high in interdependent self-construal compared to their low interdependent self-construal counterparts.

## Materials and Methods

### Participants

We used two waves of the data sets from MIDUS to test our hypotheses. This data set is composed of a nationally representative group of individuals aimed at examining age-related differences in physical and mental health. An initial survey was conducted in 1995–1996 (Wave 1) and recruited a sample of 7,108 non-institutionalized adults from the 48 contiguous states via random-digit dialing of telephone numbers. In a follow-up survey conducted in 2004–2006 (Wave 2), seventy percent of the initial sample participated again. The final sample of 4,963 respondents (females = 2,647, males = 2,316), who participated both in Wave 1 and 2 surveys, was entered in the current analyses. The ages of these participants ranged from 20 to 75 years at Wave 1 (*M* = 46.46 years, SD = 12.51) and ranged from 28 to 84 years at Wave 2 (*M* = 55.43 years, SD = 12.45).

### Measures

#### Self-Construal

Participants completed the Self-Construal scale, which consists of interdependent self-construal and independent self-construal subscales (Singelis, 1994). Interdependent and independent self-construal was assessed only at Wave 2. Each scale contained three items (“My happiness depends on the happiness of those around me,” “I often have the feeling that my relationships with others are more important than my own accomplishments,” and “It is important to listen to other’s opinions” for the interdependent self-construal subscale; “I act in the same way no matter who I am with,” “I enjoy being unique and different from others in many respects,” and “Being able to take care of myself is a primary concern for me” for the independent self-construal subscale). Participants rated the extent of their agreement with each item on a 7-point scale (1 = *strongly disagree*, 7 = *strongly agree*). Average scores of the items in each scale were computed to form separate composites for the interdependent and independent self-construal scales (*M*_inter_ = 4.72, SD = 1.13; *M*_indep_ = 5.25, SD = 1.07). Consistent with the previous literature, the two self-construal subscales were not correlated (*r* = 0.01, *p* = 0.51).

#### Loss of Child

The experience of losing a child was assessed in two ways. First, participants reported whether they have ever experienced a loss of a child. This self-report was only measured in the Wave 2 survey. There were 2,394 respondents who provided this information, and 14.2% of them (339) reported that they lost at least one child in their lifetime. While this measure is the most face valid measure of loss in the data set, it did not allow us to control for when the loss might have occurred (e.g., 30 years vs. 1 year ago). To help control for this concern, for our second measure of loss, we subtracted the number of children at Wave 2 from the number of children at Wave 1, and defined those having a negative number for this difference score as parents who had experienced child loss. There were 4,064 respondents who provided the number of children both at Wave 1 and 2, and 7.3% of these participants (364) had fewer children at Wave 2 than Wave 1.

#### Purpose in Life

Purpose in life was measured by using the purpose in life subscale of Psychological Well-Being scale (PWB; Ryff, 1989). The subscale consisted of three items [“I live life 1 day at a time and don’t really think about the future (reversed),” “Some people wander aimlessly through life, but I am not one of them,” and “I sometimes feel as if I’ve done all there is to do in life (reversed);” at Wave 1, at Wave 2], which were rated on a 7-point scale (1 = *strongly disagree*, 7 = *strongly agree*). A sum of the items was calculated and used as an indicator of purpose in life (*M* = 16.73, SD = 3.50 at Wave 1; *M* = 16.21, SD = 3.42 at Wave 2).

#### Covariates

Age, gender, level of education, income, number of children (alive), and Big Five personality from the Wave 2 data were used as covariates in our analyses. The level of education was measured by asking the highest grade of school or year of college participants completed using a 12-point scale (1 = *no school or some grade school*, 7 = 3 *or more years of college, no degree yet*, 12 = *Ph.D., MD, or other professional degree*; *M* = 7.20, SD = 2.52). The personal annual income was assessed on a 42-point scale (1 = *less than $0/loss*, 42 = *$200,000 or more*). The median income was 14 ($22,500–$24,499). The average number of children was 2.50 (SD = 1.76).

## Results

### Cross-sectional Analyses

**FIGURE 1:**
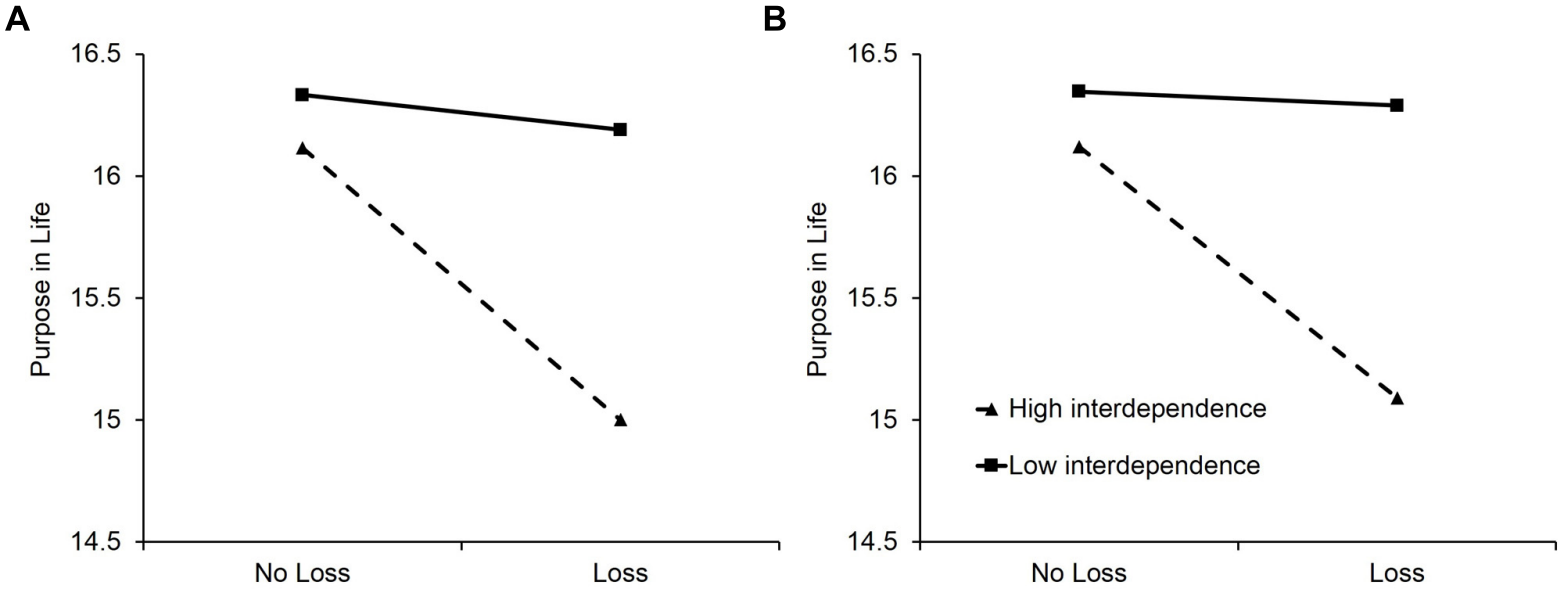
**Regression lines predicting purpose in life as a function of losing a child for individuals ±1 SD from the mean on interdependent self-construal in the cross-sectional **(A)** and longitudinal **(B)** analyses**.

We first conducted a hierarchical linear regression analysis using the self-reported loss of a child variable in a cross-sectional manner to test our hypothesis. The main effects of interdependent self-construal (centered) and the self-report item assessing losing a child (effect coded; -1 = *no loss*, 1 = *loss of child*) were entered in Step 1, and their interaction term was entered in Step 2. As presented in **Table 1**, we found that both the loss of a child (*b* = -0.31, *p* = 0.002) and interdependent self-construal (*b* = -0.17, *p* = 0.007) negatively predicted purpose in life. Importantly, however, the interaction effect was significant (*b* = -0.22, *p* = 0.01). As shown in **Figure 1A**, the experience of losing a child predicted less purpose in life for people high in interdependent self-construal (*b* = -0.56, *p* < 0.001), whereas losing a child was unrelated to purpose in life for those low in interdependent self-construal (*b* = -0.07, *p* = 0.60). This interaction pattern remained consistent even when relevant covariates (i.e., age, gender, education level, income, number of children, Big Five) were accounted for (see **Table 2**).

**Table 1:** Cross-sectional analysis.

		Purpose in life W2			
	Predictor	*B*	β	*t*	*ΔR^2^*
Step 1	LOSS INTER	-0.308 -0.167	-0.063 -0.055	-3.08^∗∗^ -2.70^∗∗^	0.007^∗∗∗^
Step 2	LOSS × INTER	-0.215	-0.071	-2.58∗	0.003∗

*A hierarchical linear regression analysis predicting purpose in life from loss of child experience, interdependent self-construal (Step 1), and interaction between loss of child and interdependent self-construal (Step 2).*

*LOSS, loss of child (-1 = *no loss*, 1 = *loss*); INTER, interdependent self-construal; W2, Wave 2.*

*∗*p* < 0.05, ∗∗*p* < 0.01, ∗∗∗*p* < 0.001.*

**Table 2:** Cross-sectional and longitudinal analyses.

		Cross-sectional analyses				Longitudinal analyses			
		Purpose in life W2				Purpose in life W2			
	Predictor	*B*	β	*t*	*ΔR^2^*	*B*	β	*t*	*ΔR^2^*
Step 1	Age Gender Income Education # Children EXTRA NEURO OPEN CONS AGREE PIL W1	-0.034 0.223 0.016 0.208 0.110 0.268 -0.446 0.379 1.385 0.309	-0.123 0.034 0.056 0.158 0.059 0.047 -0.085 0.061 0.181 0.047	-5.03∗∗∗ 1.42 2.27∗ 7.00∗∗∗ 2.72∗∗ 1.74^†^ -3.75∗∗∗ 2.34∗ 7.99∗∗∗ 1.82^†^	0.137∗∗∗	-0.023 0.131 0.013 0.095 0.065 0.328 -0.304 0.401 0.887 0.074 0.347	-0.080 0.020 0.048 0.072 0.032 0.056 -0.057 0.064 0.117 0.011 0.356	-4.17∗∗∗ 1.04 2.38∗ 3.91∗∗∗ 1.83^†^ 2.60∗∗ -3.15∗∗ 3.10∗∗ 6.25∗∗∗ 0.53 20.04∗∗∗	0.249∗∗∗
Step 2	LOSS INTER	-0.054 -0.067	-0.011 -0.023	-0.52 -1.06	0.001	-0.175 -0.065	-0.029 -0.022	-1.68^†^ -1.28	0.001
Step 3	LOSS × INTER	-0.170	-0.057	-2.00∗	0.002∗	-0.250	-0.084	-2.75∗∗	0.002∗∗

*Hierarchical linear regression analyses predicting purpose in life from covariates (Step 1), loss of child experience, interdependent self-construal (Step 2), and interaction between loss of child and interdependent self-construal (Step 3).*

*Gender: Female = 0, Male = 1; #Children, Number of Children; EXTRA, Extraversion; NEURO, Neuroticism; OPEN, Openness; CONS, Consciousness; AGREE, Agreeableness; PIL, purpose in life; LOSS, loss of child (-1 = *no loss*, 1 = *loss*); INTER, interdependent self-construal; W1, Wave 1; W2, Wave 2.*

*^†^*p* < 0.10, ∗*p* < 0.05, ∗∗*p* < 0.01, ∗∗∗*p* < 0.001.*

Next, we ran the same hierarchical linear regression analysis, substituting independent self-construal for interdependent self-construal, that is, entering the main effect terms of independent self-construal (centered) and the self-report of losing child (effect coded) in Step 1, and their interaction term in Step 2. Again, loss of a child predicted lower purpose in life (*b* = -0.31, *p* = 0.002), but independent self-construal (*b* = 0.04, *p* = 0.50) and the interaction term (*b* = 0.11, *p* = 0.23) did not significantly predict feelings of purpose.

### Longitudinal Analyses

Using the change in number of child between Wave 1 and Wave 2 as a proxy for the experience of losing child, we tested our hypothesis again in a longitudinal manner. We ran a similar hierarchical linear regression analysis by entering purpose in life at Wave 1 (centered) in Step 1, as a covariate, the change in number of child (effect coded; -1 = *no loss*, 1 = *loss of child*) in Step 2, and its interaction with interdependent self-construal in Step 3 as predictors of purpose in life at Wave 2. As presented in **Table 3**, we found that purpose at Wave 1 (Step 1), losing a child, and interdependent self-construal (Step 2) each significantly predicted purpose in life at Wave 2 (*b* = 0.43, *p* < 0.001; *b* = -0.27, *p* = 0.004; *b* = -0.14, *p* = 0.003, respectively). As predicted, however, these main effects were qualified by a significant interaction effect (*b* = -0.22, *p* = 0.008). The pattern was consistent with the cross-sectional findings (see **Figure 1B**). Simple slope tests revealed that the experience of losing child predicted lowered purpose in life for parents high in interdependent self-construal (*b* = -0.51, *p* < 0.001), whereas the same experience was not related to purpose in life for those low in interdependent self-construal (*b* = -0.03, *p* = 0.83). Results were unchanged when other relevant covariates were included in the analysis (see **Table 2**).

**Table 3:** Longitudinal analysis.

		Purpose in life W2			
	Predictor	*B*	β	*t*	*ΔR^2^*
Step 1	PIL W1	0.432	0.446	28.28∗∗	0.199∗∗
Step 2	LOSS INTER	-0.273 -0.141	-0.045 -0.046	-2.86∗ -2.95∗	0.004∗∗
Step 3	LOSS × INTER	-0.215	-0.071	-2.65∗	0.002∗

*A hierarchical linear regression analysis predicting purpose in life at Wave 2 from purpose in life at Wave 1 (Step 1), loss of child experience, interdependent self-construal (Step 2), and interaction between loss of child and interdependent self-construal (Step 3).*

*PIL, purpose in life; LOSS, loss of child (-1 = *no loss*, 1 = *loss*); INTER, interdependent self-construal; W1, Wave 1; W2, Wave 2.*

*∗*p* < 0.01, ∗∗*p* < 0.001.*

We performed the same analyses, substituting independent self-construal for interdependent self-construal. The main effects of purpose in life at Wave 1, losing a child, and independent self-construal were significant predictors of purpose in life at Wave 2 (*b* = 0.43, *p* < 0.001; *b* = -0.29, *p* = 0.003; *b* = 0.11, *p* = 0.022, respectively). However, the interaction term failed to predict purpose in life (*b* = -0.04, *p* = 0.65).

### Additional Analyses

We also conducted the additional analyses to examine how interdependent self-construal and parental bereavement predict other well-being variables (i.e., subjective well-being and depression) that were available in MIDUS. The results of these analyses did not reveal consistent patterns, indicating that the interactive effect between interdependent self-construal and parental bereavement existed only with regard to purpose in life (see Supplementary Material for tables depicting these null effects).

## Discussion

These findings support our hypothesis that loss of a child erodes one’s sense of purpose in life and that this impact is particularly pronounced for those with an interdependent self-construal. In both cross-sectional and longitudinal analyses, we found that experiencing loss of a child significantly lowered purpose in life among highly interdependent parents, whereas bereavement did not affect purpose in life among parents low in interdependent self-construal. In both analyses, independent self-construal did not moderate the effect of loss of children on purpose in life. These findings are consistent with extant research on hedonic adaptation demonstrating that important individual differences may bear on the restoration of psychological equanimity after the experience of a negative life event (e.g., Lucas et al., 2003; Luhmann et al., 2012).

Although our results found that purpose in life among parents low in interdependent self-construal did not seem to be affected by a loss of a child, we do not suggest that these people are immune to negative responses from these types of traumatic events. Rather, we believe that this indicates that they may be better at “bouncing back” from the trauma than highly interdependent parents. They may, for example, have better coping strategies that help them reconstruct purpose in life (e.g., they may find it easier to focus on other domains in life such as work). Of course, it is also possible that the initial experience of bereavement is stronger for highly interdependent individuals (cf., Lucas et al., 2003). The adaptation process might be homogenous for all parents but, because of this initial difference, those with interdependent self-construal may take much longer to regain their sense of purpose. Future research needs to explore the mechanisms underlying the different levels of purpose in life between bereaved parents high and low in interdependent self-construal.

Interestingly, independent self-construal did not affect purpose in life among bereaved parents. This finding suggests that experiencing parental bereavement life events is uniquely associated with interdependent self-construal rather than independent self-construal, which is consistent with prior research demonstrating that the dimensions of independent and interdependent self-construals are orthogonal and thus can coexist in individuals (e.g., Singelis, 1994). However, it is possible that other types of personal loss, that have more individualistic implications (e.g., losing one’s eyesight), might relate to purpose in life more strongly for people high in independent self-construal. Future research should investigate whether the various types of personal loss uniquely interact with independent and interdependent self-construal to predict a sense of purpose.

Hedonic well-being is described as a subjective state of feeling pleasure and satisfied with one’s life, whereas eudaimonic well-being is defined as a state of human flourishing that is achieved from pursuing goals expressing one’s true self and giving purpose and meaning to his life (Ryan and Deci, 2001). These two aspects of well-being often operate in tandem (e.g., King et al., 2006) but are also theoretically and empirically distinct (e.g., Baumeister et al., 2013). Previous literatures on adaptation following critical life events primarily focus on changes in hedonic aspects of happiness (i.e., subjective well-being; Lucas et al., 2003; Luhmann et al., 2012). However, eudaimonic aspects of happiness are also influenced by various life events (Waterman, 2007; Durkin and Joseph, 2009; Uchida et al., 2014). Our research highlights purpose in life as one particular dimension of eudaimonic well-being that is affected by the loss of a child. Future research should examine how self-construal and specific types of trauma uniquely detract from hedonic and eudaimonic sources of happiness.

The current findings have implications for cultural psychology by showing that the impact of parental bereavement on purpose in life is more pronounced for interdependent people than independent people. An obvious limitation is that we only compared interdependent and independent people within the same culture. Future research should examine whether the same pattern of results emerges in direct cross-cultural comparisons. For example, is it possible that the loss of a child is more traumatic for people from Eastern cultures compared to Western cultures? Or, perhaps people from Eastern cultures have other types of coping mechanisms that help them regain a sense of purpose following the loss of a child? These possibilities remain to be addressed by future research.

## Conclusion

Our findings demonstrate that the loss of a child threatens parents’ sense of purpose and that it may be particularly difficult for highly interdependent parents to rediscover meaningful goal pursuits after such tragedy. It is our hope that future research will uncover the underlying mechanisms driving this effect and the variables that help highly interdependent people cope with the loss of a child.

## Author Contributions

JK and JH developed the research hypothesis. JK performed analysis, interpreted data, and prepared manuscript. JH supervised data analysis and helped data interpretation and manuscript preparation.

## Conflict of Interest Statement

The authors declare that the research was conducted in the absence of any commercial or financial relationships that could be construed as a potential conflict of interest.
