# Supplementary material for: Parental bereavement and the loss of purpose in life as a function of interdependent self-construal
Source: Front Psychol. 2015 Jul 27;6:1078. doi: 10.3389/fpsyg.2015.01078 (PMC4515542; doi:10.3389/fpsyg.2015.01078)
Supplement: Supplementary file 1 [file Table_1.PDF]

frontiers

Supplementary Material

Parental bereavement and the loss of purpose in life as a function of interdependent self-construal

Jinhyung Kim*, Joshua A. Hicks

*** Correspondence:** Jinhyung Kim: jhkim82@tamu.edu

1. Supplementary Analyses

We conducted a series of the analyses to examine how interdependent self-construal and parental bereavement predict two well-being measures: subjective well-being (SWB) and depression. In MIDUS, SWB was measured through a sum of affective balance (i.e., positive affect - negative affect, e.g., cheerful and nervous, respectively) and life satisfaction (satisfaction with life overall, work, health, and relationship with spouse/partner), and depression was assessed using 7 items asking about depressed affect (e.g., “People sometimes feel down on themselves, no good, or worthless. During that two-week period, did you feel this way?”). As presented in Tables below, the results did not reveal consistent interaction effects of interdependent self-construal and parental bereavement on SWB (see Supplementary Tables 1 through 3) and depression (see Supplementary Tables 4 through 6).

1.1. Supplementary Tables

Supplementary Table 1. Cross-sectional analysis. A hierarchical linear regression analysis predicting subjective well-being from loss of child experience, interdependent self-construal (Step 1), and interaction between loss of child and interdependent self-construal (Step 2)

		SWB W2			
	Predictor	*B*	β	*t*	ΔR^2
Step 1	LOSS	-.119	-.039	-1.90[†]	
	INTER	-.044	-.024	-1.14	.002[†]
Step 2	LOSS × INTER	-.037	-.020	-.71	.000

Note. SWB = Subjective Well-Being; LOSS = Loss of Child (-1 = *no loss*, 1 = *loss*); INTER = Interdependent Self-Construal; W2 = Wave 2.
[†] $p < .10$.

Supplementary Table 2. Longitudinal analysis. A hierarchical linear regression analysis predicting subjective well-being at Wave 2 from subjective well-being at Wave 1 (Step 1), loss of child experience, interdependent self-construal (Step 2), and interaction between loss of child and interdependent self-construal (Step 3)

	Predictor	SWB W2 *B*	β	*t*	ΔR^2
Step 1	SWB W1	.591	.585	40.64^{**}	$.342^{**}$
Step 2	LOSS	.010	.003	.18	
	INTER	-.056	-.030	-2.07^{*}	.001
Step 3	LOSS × INTER	-.088	-.046	$-1.88^{\dagger}$	$.001^{\dagger}$

Note. SWB = Subjective Well-Being; LOSS = Loss of Child (-1 = *no loss*, 1 = *loss*); INTER = Interdependent Self-Construal; W1 = Wave 1; W2 = Wave 2.
† $p < .10$. * $p < .05$. ** $p < .001$.

Supplementary Table 3. Cross-sectional and longitudinal analyses. Hierarchical linear regression analyses predicting subjective well-being from covariates (Step 1), loss of child experience, interdependent self-construal (Step 2), and interaction between loss of child and interdependent self-construal (Step 3)

		Cross-sectional analyses				Longitudinal analyses			
		SWB W2				SWB W2			
	Predictor	*B*	β	*t*	ΔR^2	*B*	β	*t*	ΔR^2
Step 1	Age	.026	.149	7.28^{***}		.022	.119	7.49^{***}	
	Gender	-.015	-.004	-.18		-.036	-.009	-.55	
	Income	.014	.078	3.78^{***}		.014	.077	4.62^{***}	
	Education	.058	.070	3.74^{***}		.054	.065	4.36^{***}	
	# Children	-.014	-.012	-.65		-.015	-.012	-.83	
	EXTRA	1.022	.281	12.57^{***}		.751	.202	11.35^{***}	
	NEURO	-1.401	-.422	-22.41^{***}		-.919	-.273	-17.29^{***}	
	OPEN	-.117	-.030	-1.37		-.127	-.032	-1.19^{*}	
	CONS	.788	.163	8.61^{***}		.675	.140	9.14^{***}	
	AGREE	-.131	-.032	-1.46	$.405^{***}$	-.120	-.028	-1.66^{*}	
	SWB W1					.373	.367	23.27^{***}	$.495^{***}$
Step 2	LOSS	-.178	-.058	-3.27^{**}		-.055	-.014	-1.02	
	INTER	.011	.006	-.32	$.004^{**}$	-.011	-.006	-.43	.000
Step 3	LOSS × INTER	-.011	-.006	-.24	.000	-.059	-.032	-1.25	.000

Note. Gender: Female = 0, Male = 1; # Children = Number of Children; EXTRA = Extraversion; NEURO = Neuroticism; OPEN = Openness; CONS = Consciousness; AGREE = Agreeableness; SWB = Subjective Well-Being; LOSS = Loss of Child (-1 = *no loss*, 1 = *loss*); INTER = Interdependent Self-Construal; W1 = Wave 1; W2 = Wave 2.
* $p < .10$. ** $p < .01$. *** $p < .001$.

Supplementary Table 4. Cross-sectional analysis. A hierarchical linear regression analysis predicting depression from loss of child experience, interdependent self-construal (Step 1), and interaction between loss of child and interdependent self-construal (Step 2)

	Predictor	Depression W2 *B*	β	*t*	ΔR^2
Step 1	LOSS	.012	.005	.25	
	INTER	.003	.002	1.04	.000
Step 2	LOSS × INTER	-.033	-.023	-.84	.000

Note. LOSS = Loss of Child (-1 = *no loss*, 1 = *loss*); INTER = Interdependent Self-Construal; W2 = Wave 2.

Supplementary Table 5. Longitudinal analysis. A hierarchical linear regression analysis predicting depression at Wave 2 from depression at Wave 1 (Step 1), loss of child experience, interdependent self-construal (Step 2), and interaction between loss of child and interdependent self-construal (Step 3)

		Depression W2			
	Predictor	*B*	β	*t*	ΔR^2
Step 1	DEP W1	.409	.213	4.14^{*}	$.045^{*}$
Step 2	LOSS	.164	.040	.79	
	INTER	-.025	-.011	-.21	.002
Step 3	LOSS × INTER	-.018	-.008	-.09	.000

Note. DEP = Depression; LOSS = Loss of Child (-1 = *no loss*, 1 = *loss*); INTER = Interdependent Self-Construal; W1 = Wave 1; W2 = Wave 2.
* $p < .001$.

Supplementary Table 6. Cross-sectional and longitudinal analyses. Hierarchical linear regression analyses predicting depression from covariates (Step 1), loss of child experience, interdependent self-construal (Step 2), and interaction between loss of child and interdependent self-construal (Step 3)

		Cross-sectional analyses				Longitudinal analyses			
		Depression W2				Depression W2			
	Predictor	*B*	β	*t*	ΔR^2	*B*	β	*t*	ΔR^2
Step 1	Age	-.012	-.087	-3.50^{***}		-.040	-.152	-2.67^{**}	
	Gender	-.291	-.090	-3.76^{***}		-.414	-.073	-1.25	
	Income	-.008	-.059	-2.33^{*}		-.049	-.206	-3.34^{**}	
	Education	-.011	-.018	-.79		.014	.013	.29	
	# Children	.016	.017	.78		-.005	-.003	-.06	
	EXTRA	-.391	-.140	-5.15^{***}		-.565	-.123	$-1.80^{\dagger}$	
	NEURO	.611	.240	10.44^{***}		1.002	.248	4.32^{***}	
	OPEN	.228	.075	2.85^{**}		.871	.180	2.72^{**}	
	CONS	-.091	-.025	-1.07		-.095	-.016	-.28	
	AGREE	.191	.060	2.29^{*}	$.109^{***}$	.253	.046	.72	
	DEP W1					.304	.159	2.96^{**}	$.206^{***}$
Step 2	LOSS	.033	.014	.64		.377	.093	$1.77^{\dagger}$	
	INTER	-.021	-.015	-.68	.000	-.035	-.015	-.29	.008
Step 3	LOSS × INTER	-.013	-.009	-.30	.000	-.175	-.076	-.88	.002

Note. Gender: Female = 0, Male = 1; # Children = Number of Children; EXTRA = Extraversion; NEURO = Neuroticism; OPEN = Openness; CONS = Consciousness; AGREE = Agreeableness; DEP = Depression; LOSS = Loss of Child (-1 = *no loss*, 1 = *loss*); INTER = Interdependent Self-Construal; W1 = Wave 1; W2 = Wave 2.
† $p < .10$. * $p < .05$. ** $p < .01$. *** $p < .001$.
